# Real-Time Trajectory Publishing in Mobile Crowdsensing Under Local Differential Privacy

**DOI:** 10.3390/e28101052

**Published:** 2026-09-24

**Authors:** Hongyi Zhang, Liquan Chen, Huiyu Fang, Jianchang Lai, Xinyuan Sun

**Affiliations:** 1 School of Cyber Science and Engineering, Southeast University, Nanjing 210096, China; hyzhang1276@seu.edu.cn (H.Z.); fhy837637215@gmail.com (H.F.); jclai@seu.edu.cn (J.L.); yaphetsun98@gmail.com (X.S.); 2 Purple Mountain Laboratories, Nanjing 211189, China

**Keywords:** local differential privacy, real-time trajectory publishing, mobile crowdsensing

## Abstract

Mobile crowdsensing (MCS) leverages the sensing capabilities of mobile devices carried by a vast number of participants to accomplish large-scale urban sensing tasks. In MCS systems, participants continuously generate trajectory streams while uploading sensing data, which raises severe risks of location privacy leakage. Local differential privacy (LDP) provides strong guarantees for user-side privacy protection. However, existing approaches for real-time trajectory publishing in MCS remain inadequate; they fail to fully leverage the continuously evolving spatial density and temporal structures within trajectory streams, which leads to suboptimal utility and distorted spatiotemporal representations under strict privacy constraints. To address this issue, we propose AdaGrid-Syn, a two-layer adaptive grid framework for real-time trajectory publishing under LDP. AdaGrid-Syn dynamically adjusts spatial granularity based on changes in density distributions and transition patterns. In addition, we develop a new adaptive privacy budget allocation strategy that distributes privacy budgets according to the temporal variation of Markov transition matrices, enabling better capture of time-sensitive behavioral characteristics. Experimental results demonstrate that our framework significantly outperforms existing approaches.

## 1. Introduction

Mobile crowdsensing (MCS) has emerged as an effective paradigm for large-scale urban sensing, leveraging the sensing capabilities of mobile devices carried by a vast number of participants to accomplish various applications, including traffic hotspot identification [1], congestion control [2] and traffic prediction [3]. In MCS systems, participants continuously generate trajectory streams while uploading sensing data. Compared with traditional offline data, real-time trajectory streams in MCS capture fine-grained spatial density variations and behavioral drifts, supporting more accurate and time-sensitive spatiotemporal analytics. While continuously collected data facilitates many valuable services, it also increases privacy risks [4,5,6]: short sequences of consecutively exposed locations can reveal users’ home addresses, commuting routines, and personal preferences, leading to severe privacy breaches.

Differential privacy (DP) [7] has become the de facto standard for privacy protection and is widely applied to various data publishing tasks. However, DP assumes a trustworthy aggregator, and users remain at risk if the aggregator becomes curious or acts for commercial interests. To address this limitation, researchers proposed local differential privacy(LDP), where data are perturbed locally before being sent to the server. LDP [8] has been applied to frequency estimation [9], frequent sequence mining [10], and mean value statistics [11], and has been deployed by Google [12], Apple [13], and Microsoft [14].

Existing LDP-based trajectory publishing approaches [15,16,17,18] mainly discretize geographic space and perturb the entire region to extract key mobility patterns under privacy constraints. These methods are primarily designed for static historical trajectories and often depend on historical characteristics or external knowledge (e.g., POI categories), limiting their ability to support truly real-time processing.

To the best of our knowledge, RetraSyn [19] is the only existing approach that satisfies LDP requirements in real-time trajectory publishing scenarios applicable to MCS. RetraSyn discretizes the geographic space using a fixed uniform grid, perturbs and aggregates users’ transition events at each timestamp to construct a global mobility model, and synthesizes trajectories that are consistent with the current spatiotemporal patterns via Markov model. However, a fixed uniform grid is inadequate for modeling the highly imbalanced spatial density commonly observed in practical MCS trajectory data. Hotspot regions and sparse areas vary significantly in both scale and structural complexity. A uniform partition is too coarse to capture detailed mobility patterns in high-density areas and exacerbates LDP-induced noise in low-density regions, leading to unstable estimates and inconsistent utility across spatial zones.

This paper proposes a new real-time trajectory publishing framework AdaGrid-Syn for mobile crowdsensing, which aims to improve the utility of synthesized trajectories for downstream MCS services while satisfying local differential privacy constraints. We position AdaGrid-Syn as a privacy-preserving trajectory publishing layer between mobile sensing participants and downstream MCS applications. Before transmitting trajectory streams to the MCS platform, each participant locally perturbs the trajectory-related information. The platform then aggregates the locally perturbed reports and continuously generates synthetic trajectory streams accordingly. The resulting synthetic trajectories can serve as a privacy-preserving mobility data source for downstream MCS services or external data analysts for analysis.

Specifically, AdaGrid-Syn integrates adaptive spatial grid structure, mobility model construction, dynamic privacy budget scheduling and real-time trajectory synthesis into a unified LDP-compliant framework. In particular, the framework employs a two-layer adaptive spatial grid structure that maintains global spatial consistency while performing localized refinement according to real-time density variations and perturbation uncertainty, enabling effective handling of pronounced spatial heterogeneity in trajectory streams without requiring global prior knowledge. In addition, AdaGrid-Syn constructs a time-evolving Markov model over the set of leaf cells and dynamically allocates privacy budgets based on the magnitude of structural changes in the Markov model. This design allows more reliable structure estimation during periods of significant mobility variation, while reducing unnecessary privacy expenditure during relatively stable phases.

Based on the above design, the main contributions of this work are summarized as follows:

1. We propose a two-layer adaptive spatial grid modeling approach for real-time trajectory streams in MCS, which dynamically adjusts spatial resolution to provide fine-grained modeling in hotspot regions.
2. We design an adaptive privacy budget allocation strategy driven by variations in the Markov transition structure. The proposed strategy enables fine-grained budget scheduling under a limited privacy budget.
3. We perform a thorough experimental evaluation across various synthetic and real-world datasets. The results consistently show that our framework provides enhanced utility while maintaining privacy guarantees.

## 2. Related Work

With the continuous generation of large-scale trajectory stream data in mobile crowdsensing (MCS), protecting sensitive trajectory privacy while maintaining utility has become an important research topic [1,2,3,20]. To systematically discuss related studies, we review three categories: (1) DP/LDP in data streams, (2) privacy-preserving location and trajectory mechanisms in MCS, (3) privacy-preserving trajectory publishing and synthesis.

### 2.1. DP/LDP in Data Streams

Beyond trajectories, privacy-preserving continuous data stream publishing in MCS has drawn significant attention. In infinite data streams, two classical privacy granularities are commonly considered. Event-level privacy protects individual data at each timestamp, which enables low-noise perturbation but leads to unbounded privacy loss when events are continuously released over time. In contrast, user-level privacy aims to protect an entire user’s data stream, yet it cannot be directly applied to infinite data streams. The notion of *w*-event privacy for infinite streams was proposed in [21] to balance user-level and event-level privacy. Using sliding-window methods, they introduce two mechanisms—Budget Distribution (BD) and Budget Absorption (BA). *w*-event privacy has been further studied under DP [22,23,24,25,26]. In the context of streaming data under LDP [27], proposes a pattern-aware sampling method based on piecewise linear approximation (PLA) with local adaptive perturbation, though it implements metric-based LDP rather than pure LDP. Further improvements are made in [28], which employs Kalman filtering for post-processing. The authors of [29] adaps BD and BA into LDP-compliant variants LBD and LBA, and proposes two population-splitting mechanisms. The authors of [30] propose an exponential mechanism-based approximation strategy for local differential privacy that selects the optimal historical release instead of direct perturbation. Schäler et al. [31] provide a systematic study of *w*-event DP frameworks, showing differences in error, latency, and task suitability. However, the above methods do not fully exploit contextual information for spatiotemporal modeling, and they cannot be directly applied to the trajectory release task.

### 2.2. Privacy-Preserving Location Mechanisms in MCS

Privacy preservation of location information has been extensively studied across multiple stages of the MCS workflow, including task allocation, sensing-data collection, and data aggregation. Wang et al. [32] introduce differential location privacy into sparse MCS by obfuscating participants’ sensing locations before reporting and adapting downstream inference to reduce utility loss. For privacy-preserving task allocation [33], applies differential geo-obfuscation to hide precise participant locations while retaining sufficient spatial information. Liu et al. [34] locally perturbs both locations and sensed values and enables the platform to recover aggregate sensing statistics, while Personalized LDP mechanisms have also been investigated to accommodate heterogeneous privacy preferences among MCS participants [35]. Gao et al. [36] point out that independently protecting individual locations may still expose trajectory-level features when multiple reports from the same participant are correlated over time. Their trajectory-obfuscation mechanism extracts representative stay points and perturbs them using differential-privacy-based mechanisms before releasing the obfuscated locations.

### 2.3. Privacy-Preserving Trajectory Publishing and Synthesis

In trajectory privacy protection, early research was primarily based on the centralized differential privacy (central DP) model, assuming a trusted data aggregator that can access raw trajectories and inject noise at the server side. Representative approaches include prefix-tree and hierarchy-based frameworks such as DPT [37] and its extensions [38,39], which build multi-level spatial structures and add noise to prefix counts to reconstruct synthetic trajectories, preserving density distributions, origin–destination patterns, and canonical travel behaviors. AdaTrace [40] designs an attack-resilient synthesizer balancing utility and adversarial robustness. SafePath [41] builds a noisy prefix tree with generalization hierarchies to efficiently publish differentially private trajectories, addressing the challenge of high-dimensional sparse data. PrivTrace [42] employs adaptive strategies to select first-order and second-order Markov models, achieving better utility than prior work. QCLM-Lowpass [43] proposes a location correlation estimation method based on the location increment to offer a better balance between the ability to resist correlation-based attacks and data availability.

However, DP inherently relies on a trustworthy aggregator, which poses risks when data server is untrusted or vulnerable to compromise—a common concern in MCS systems where the platform may be untrustworthy. Consequently, a series of LDP-based trajectory publishing methods emerged. NGRAM [15] perturbs overlapping n-grams locally using the exponential mechanism and external auxiliary knowledge (e.g., POIs, business hours), and reconstructs trajectories server-side. LDPTrace [16] extracts key features (length distribution, Markov models, start/end distributions) locally and estimates global mobility patterns server-side. LDPTP [18] captures popular regions and spatial similarities to generate privacy-preserving mobility patterns with improved utility. To address the trade-off between privacy and data utility in trajectory data publishing, WF-LDPSR [44] introduces a water-filling-based local differential privacy mechanism that adaptively allocates privacy budgets and employs user set sampling to improve data availability by 84.92%.

Recently, RetraSyn [19] extends LDP trajectory synthesis to trajectory streaming, which is particularly relevant for real-time MCS applications. At each timestamp, RetraSyn updates a global mobility model using perturbed transitions under *w*-event LDP constraints, enabling online synthesis. It introduces dynamic mobility updates and budget adaptation to capture evolving patterns and maintain utility for diverse downstream tasks. However, due to the adoption of a fixed grid partition, it is difficult for RetraSyn to capture fine-grained mobility patterns.

Building upon these studies, this paper proposes a trajectory synthesis framework for trajectory streams, which seeks to better capture spatiotemporal evolution characteristics and improve utility under LDP.

## 3. Preliminaries

In this section we first introduce the basic concepts used throughout this work and then formalize our problem.

### 3.1. Local Differential Privacy

Local differential privacy (LDP) requires each user to perturb their data independently and subsequently report the noisy data to the curator for aggregation.

**Definition** **1.** *ϵ-Local Differential Privacy. Let Ψ:X→Y be a randomized mechanism. For any two possible inputs $x_{1},x_{2}\in X$ and any output y∈Y, if*

(1) $$\mathrm{Pr}\left[\Psi \left(x_{1}\right)=y\right]\leq e^{\epsilon }\mathrm{Pr}\left[\Psi \left(x_{2}\right)=y\right],$$

*then *Ψ* is said to satisfy ϵ-local differential privacy (ϵ-LDP), where ϵ>0 is the privacy budget. A smaller ϵ implies stronger privacy guarantee.*

As with DP, LDP enjoys the desired properties of sequential composition and post-processing [45,46].

**Theorem** **1.** *Sequential Composition. Let $\Psi_{i}$(1≤i≤n) be a local differential privacy mechanism satisfying $\epsilon_{i}$-LDP for each i=1,…,n. Then the mechanism $M:X\to Y_{1}\times \cdots \times Y_{n}$ defined by the sequential application of $M_{1},\ldots ,M_{n}$ satisfies ϵ-LDP with $\epsilon =\sum_{i=1}^{n}\epsilon_{i}$.*

**Theorem** **2.** *Post-Processing. Let M:X→Y be an ϵ-locally differentially private mechanism, and let $f:Y\to Y^{\prime }$ be an arbitrary randomized mapping. Then the composed mechanism $f\circ M:X\to Y^{\prime }$ also satisfies ϵ-LDP.*

The frequency oracle (FO) is a canonical task in LDP protocols: given a value drawn from a discrete domain, the curator aims to estimate its frequency without accessing the raw data. In this work, we adopt Optimized Unary Encoding (OUE) [37] as the FO protocol for achieving frequency estimation under LDP. OUE consists of three steps: encoding, perturbing, and aggregation.

1. **Encoding.** Given a user’s true input *x* from a domain of size *d*, the user encodes it into a binary vector $V\in {\left\{0,1\right\}}^{d}$, where only the *x*-th entry is set to 1, i.e., V[i]=1(i=x).
2. **Perturbing.** Each bit of *V* is independently randomized to produce the perturbed vector $\hat{V}$. For the *i*-th position, the probability that the reported bit equals 1 is
  (2) $$\mathrm{Pr}\left[\hat{V}\left[i\right]=1\right]=\left\{\begin{array}{cc} \frac{1}{2}, & V[i]=1,\\ \frac{1}{e^{\epsilon }+1}, & V[i]=0, \end{array}\right.$$
  where ϵ is the privacy budget used for this report. This randomized response ensures that $\hat{V}$ satisfies ϵ-LDP.
3. **Aggregation.** The curator aggregates all reported noise vectors. Let g(x) denote the number of users whose reported vector has $\hat{V}\left[x\right]=1$, and let *n* be the total number of users. Since the perturbation introduces systematic bias, the curator applies a linear correction. Let $q=\frac{1}{e^{\epsilon }+1},$ then the adjusted estimator is given by
  (3) $$\tilde{g}\left(x\right)=\frac{g(x)-nq}{\frac{1}{2}-q}.$$
  This estimator is unbiased, satisfying $E\left[\tilde{g}\left(x\right)\right]=nf_{x}$, where $f_{x}$ is the true frequency of value *x*. Moreover, its variance is
  (4) $$\mathrm{Var}\left[\tilde{g}\left(x\right)\right]=n\cdot \frac{4e^{\epsilon }}{{\left(e^{\epsilon }-1\right)}^{2}}.$$

### 3.2. w-Event LDP

*w*-event LDP is a privacy notion proposed for protecting users in infinite data streams. While classical LDP protects either a single report (event-level privacy) or the entire user history (user-level privacy), *w*-event LDP offers a more flexible and controllable intermediate guarantee by ensuring privacy within any sliding window of size *w*. This model restricts the total privacy leakage that may occur in any window of *w* consecutive reports, thereby enabling practical privacy protection for continuous data streams such as trajectory streams.

A data stream is modeled as an ordered sequence $S_{\left[t\right]}=\left\{c_{1},c_{2},\ldots \right\}$, where $c_{i}$ denotes the value reported at timestamp *i*. The corresponding stream prefix at time *t* is defined as $S_{\left[t\right]}=\left\{c_{1},c_{2},\ldots ,c_{t}\right\}$, which represents all observations accumulated up to time *t*. Before introducing w-event LDP, we first recall the notion of w-neighboring, which characterizes how two stream prefixes may differ within a bounded temporal window.

**Definition** **2.** *w-Neighboring. Let w be a positive integer. Two stream prefixes $S_{\left[t\right]}=\left(c_{1},\ldots ,c_{t}\right)$ and $S_{\left[t\right]}^{\prime }=\left(c_{1}^{\prime },\ldots ,c_{t}^{\prime }\right)$ are said to be* w-neighboring *if they satisfy the following:*

*1.*  *For every index i∈[t] such that $c_{i}\neq c_{i}^{\prime }$, the two events $c_{i}$ and $c_{i}^{\prime }$ are neighboring events.*
*2.*  *Let $I=\{\;i\in \left[t\right]|c_{i}\neq c_{i}^{\prime }\;\}$ be the set of all positions where the two prefixes differ. If I≠⌀, denote $i_{1}=\min I$ and $i_{2}=\max I$. The prefixes are w-neighboring if the span of all differing events satisfies the window constraint: $i_{2}-i_{1}+1\leq w.$*

**Definition** **3.** *w-event LDP [28]. Let Ψ be a randomized algorithm that takes as input a stream prefix $T_{t}=\left\{c_{1},\ldots ,c_{t}\right\}$. We say that Ψ satisfies w-event ϵ-LDP if, for any two w-neighboring prefixes $T_{t}$ and $T_{t}^{\prime }$ and for any output y, the following holds:*

(5) $$\mathrm{Pr}\left[\Psi \left(T_{t}\right)=y\right]\;\leq \;e^{\epsilon }\mathrm{Pr}\left[\Psi \left(T_{t}^{\prime }\right)=y\right].$$

Intuitively, w-event LDP limits the cumulative privacy leakage that may occur within any window of *w* consecutive reports. When w=1, the notion coincides with classical event-level LDP. As *w* approaches the full length of a finite stream, the guarantee gradually strengthens toward user-level privacy. This sliding-window interpretation makes *w*-event LDP particularly suitable for modeling privacy in continuous data streams, where different time intervals may have varying levels of sensitivity.

### 3.3. Problem Statement

We consider trajectory streams generated by mobile users in an MCS environment. During the sensing process, each users may continuously generate location information that reflects her mobility over time. In this work, we focus specifically on the privacy-preserving publication of such trajectory streams, rather than on the complete MCS workflow.

To protect trajectory privacy, the data curator cannot directly collect or store the raw trajectory streams. Instead, each user locally perturbs their location-related information before reporting it to the curator. The objective is to design an online release mechanism Ψ that takes the original trajectory streams T as input and outputs a dynamic synthetic trajectory database $T_{\mathrm{syn}}$. The mechanism Ψ is required to satisfy *w*-event ϵ-LDP. The generated synthetic trajectory stream should preserve the key statistical characteristics of the original data, so as to support downstream MCS tasks such as traffic monitoring, environmental sensing, and urban analytics.

## 4. Our Proposal

### 4.1. Overview

AdaGrid-Syn targets the real-time trajectory publishing problem under the *w*-event LDP constraint by continuously updating the spatial discretization structure and mobility behavior patterns, and synthesizing trajectory points that are consistent with real movement trends over a dynamic spatial domain. In this way, privacy protection is achieved while preserving the capability to capture spatiotemporal structures. AdaGrid-Syn is organized around three core modules: density-driven two-layer adaptive grid structure, mobility model construction, and real-time trajectory synthesis. In addition, the system incorporates an enhanced adaptive privacy budget allocation mechanism to improve accuracy under the sliding-window constraint. The overall framework is illustrated in Figure 1.

***Phase 1: Two-Layer Grid Adjustment.*** At each timestamp, users locate their current positions according to the grid structure from the previous round, and report perturbed density events periodically. After aggregating the density reports, the curator recovers the access intensity of different regions and identifies spatial hotspots and sparse areas. Within each coarse cell, the system independently determines whether local refinement is needed, thereby forming a two-layer adaptive grid structure that evolves over time. The resulting set of leaf cells constitutes the spatial discretization domain at the current timestamp.

***Phase 2: Mobility Model Construction.*** After determining the current spatial domain, the curator exploits the perturbed transition reported by users at each timestamp to recover the mobility model among leaf cells and identifies the most informative transitions. Afterwards, the distribution of significant transitions is updated.

***Phase 3: Real-time Trajectory Synthesis.*** Given the latest spatial domain and transition structure, the curator performs real-time synthesis of trajectories based on the Markov model generated through the mobility model. Each trajectory selects the leaf cell of the next location according to the Markov model, and then generates a continuous spatial coordinate within that leaf cell.

***Adaptive Privacy Budget allocation:*** To enhance the temporal sensitivity of mobility model under *w*-event LDP settings, AdaGrid-Syn adopts a structure-aware privacy budget allocation mechanism that allocates more budget to time periods with significant mobility model changes.

### 4.2. Two-Layer Adaptive Grid Structure

To effectively model real-time spatial distributions in perturbed trajectory streams, we first partition the entire map into K×K fixed-size coarse cells, denoted by $C=\{c_{1},c_{2},\ldots ,c_{G}\}$, where $G=K^{2}$. The coarse grid remains fixed throughout the execution, providing a stable structural basis for all spatial statistics. AdaGrid-Syn adaptively adjusts the resolution of fine grids within each coarse cell according to the perturbed probability distribution, thereby balancing spatial resolution and noise robustness.

For any coarse cell $c_{g}\in C$, let its current refinement scale be $k_{g}\in \left\{1,\ldots ,K_{\max}\right\}$. When $k_{g}=1$, the coarse cell remains unrefined; when $k_{g}>1$, the coarse cell is partitioned into $s_{g}=k_{g}^{2}$ leaf cells.

Each leaf cell is denoted by $f_{i}\in F_{t}$, where $F_{t}$ represents the effective spatial discretization domain used by the system at time *t*. As the adaptive refinement process proceeds, the number of leaf cells varies over time, enabling the system to provide higher spatial resolution in hotspot regions while maintaining lower noise sensitivity in sparse regions.

Since trajectory hotspots do not change drastically from one timestamp to the next, and real trajectory mobility exhibits strong temporal continuity and local stability, while perturbed density estimates at a single timestamp have large variance under small privacy budgets, we choose to report density events once every *w* timestamps. Specifically, users apply the OUE mechanism with the privacy budget $\epsilon_{1}$ allocated to density events to perturb their current leaf cell location $f_{i}$ and send it to the curator. Based on the perturbed reports, the server recovers the estimated access probability of each leaf cell, denoted by ${\hat{\pi }}_{t}\left(i\right)$. The overall access probability of a coarse cell $c_{g}$ is given by $p_{g}\left(t\right)=\sum_{i\in c_{g}}{\hat{\pi }}_{t}\left(i\right)$.

To characterize the internal structural complexity of each region, we measure the dispersion of the probability distribution within each coarse cell as

(6) $$H_{g}\left(t\right)=\sum_{i\in c_{g}}{\left({\hat{\pi }}_{t}\left(i\right)-\frac{p_{g}\left(t\right)}{s_{g}}\right)}^{2}.$$

High-density regions often contain more important local patterns. Therefore, we further define an effective structural metric as

(7) $$H_{g}^{\mathrm{eff}}\left(t\right)=H_{g}\left(t\right)+\alpha_{H}\;p_{g}{\left(t\right)}^{2},$$

where $\alpha_{H}$ controls the relative importance between structural and density factors.

**Refinement Scale Selection:** The refinement level within a coarse cell determines the number of leaf cells, and thus affects the accumulation of LDP noise. For a coarse cell with refinement scale *k*, the number of leaf cells is $k^{2}$. Under OUE, the overall noise magnitude induced by independent perturbations within the coarse cell can be expressed as

(8) $${\mathrm{Var}}_{g}\left(k\right)=k^{2}\cdot \mathrm{Var}\left[\hat{\pi }\left(i\right)\right].$$

The variance of the probability estimate for a single cell under OUE is $\mathrm{Var}\left[\hat{\pi }\left(i\right)\right]=\frac{4e^{\epsilon_{1}}}{n{\left(e^{\epsilon_{1}}-1\right)}^{2}}$, where *n* denotes the number of users reporting in the timestamp and $\epsilon_{1}$ is the privacy budget for density events.

It can be observed that the noise amplification caused by refinement grows proportionally to $k^{2}$, while the improvement in structural resolution is inversely proportional to $k^{2}$. To provide a practical trade-off between the two, we define the refinement scale selection criterion for each coarse cell $c_{g}$ as

(9) $$k_{g}^{*}=\arg\min_{k\in \{1,\ldots ,K_{\max}\}}\left(\frac{H_{g}^{\mathrm{eff}}\left(t\right)}{k^{2}}+{\mathrm{Var}}_{g}\left(k\right)\right).$$

The adaptive refinement scale $k_{g}^{*}$ is obtained by minimizing the sum of the two error terms. After computing the updated $k_{g}^{*}$ for all coarse cells, the grid structure is updated and released.

### 4.3. Mobility Model Construction

At each timestamp, a user constructs a transition event $z=(f_{i}\;\to f_{j},\;\mathrm{flag})$ using the previous location $f_{i}$ and the current location $f_{j}$, where flag∈{0,1,2} indicates normal movement, entry, and exit, respectively.

To avoid excessive noise accumulation caused by high-dimensional domains and to reflect the practical observation that trajectories do not exhibit abrupt long-distance jumps within a single timestamp, the system does not allow transitions between arbitrary cells. Instead, a neighborhood N(f) is constructed over the set of leaf cells based on geometric adjacency. Specifically, two leaf cells are considered adjacent if and only if their corresponding rectangular regions have a non-empty intersection in the plane, including cases where they share a boundary edge or only a corner. The system restricts the legal transition domain to three types of transitions: self-loops $f_{i}\;\to f_{i}$, normal neighborhood transitions $f_{i}\;\to f_{j}$ where $f_{j}\in N\left(f_{i}\right)$ (non-neighbor transitions are regarded as unreachable and excluded from estimation), and entry/exit events $f_{i}\;\to f_{i}$ with flag=1,2. This neighborhood constraint allows the transition domain to adaptively vary with grid refinement, preserving fine-grained local structures while reducing the domain size from $F_{t}^{2}$ to approximately $9|F_{t}|$.

The first-order Markov assumption states that the location $T_{i+1}$ of a trajectory depends only on the previous location $T_{i}$, rather than the entire history, i.e.,

(10) $$\mathrm{Pr}\left(T_{i+1}=c|T_{1}\ldots T_{i}\right)=\mathrm{Pr}\left(T_{i+1}=c|T_{i}\right)$$

Accordingly, the statistical evolution of the trajectory sequence $\left\{T_{i}\right\}$ can be characterized by a Markov-based mobility model *M*, which drives the trajectory generation process over the discrete spatial domain.

Transition events are encoded using unary encoding and perturbed on the user side via the OUE mechanism. After receiving and aggregating the perturbed reports, the server obtains the estimated transition frequencies ${\hat{p}}_{t}\left(z\right)$ over the legal transition domain. Based on these estimates, the system constructs the mobility model over an extended state space that incorporates entry and exit states. Let $F_{t}$ denote the set of leaf cells at time *t*, and introduce two virtual states $s^{\mathrm{in}}$ and $s^{\mathrm{out}}$. The extended state space is defined as $S=\left\{s^{\mathrm{in}}\right\}\cup F_{t}\cup \left\{s^{\mathrm{out}}\right\}$.

For any two leaf cells $f_{i},f_{j}\in F_{t}$, the mobility model is constructed from the estimated transition frequencies as

(11) $$\begin{array}{cc} \; & {\hat{C}}_{t}\left(i,j\right)={\hat{p}}_{t}\left((f_{i}\;\to f_{j},0)\right),\\ \; & {\hat{C}}_{t}(s^{\mathrm{in}},i)={\hat{p}}_{t}\left((f_{i}\;\to f_{i},1)\right),\\ \; & {\hat{C}}_{t}\left(i,s^{\mathrm{out}}\right)={\hat{p}}_{t}\left((f_{i}\;\to f_{i},2)\right). \end{array}$$

All entries corresponding to illegal transitions are set to zero. The estimated Markov transition matrix ${\hat{M}}_{t}$ is then obtained by row-wise normalization of ${\hat{C}}_{t}$:

(12) $${\hat{M}}_{t}\left(i,j\right)=\frac{{\hat{C}}_{t}\left(i,j\right)}{\sum_{j^{\prime }:\left(i,j^{\prime }\right)\in D_{\mathrm{tr}}}{\hat{C}}_{t}\left(i,j^{\prime }\right)}.$$

Therefore, ${\hat{C}}_{t}$ preserves the relative transition volume across different origin states, whereas ${\hat{M}}_{t}$ describes the conditional distribution of outgoing transitions given an origin state. All other combinations that do not belong to the legal transition domain are assigned zero. After the above assignments, the mobility model is normalized row-wise. Transitions between leaf cells preserve the neighborhood constraint, while the virtual entry and exit states characterize the generation and termination of synthetic trajectories.

Since many real-world mobility flows exhibit relatively stable distributions across consecutive timestamps, we adopt a dynamic mobility update (DMU) [19] strategy to selectively update transitions that are undergoing significant changes. Let $n_{t}$ denote the number of users reporting at timestamp *t*, and let $\epsilon_{t}$ be the privacy budget allocated to transition events. If a transition $\left(f_{i}\to f_{j}\right)$ is updated at time *t* using the current perturbed statistics, the expected error introduced by the OUE mechanism can be expressed as

(13) $${\mathrm{Err}}_{\mathrm{upd}}=\frac{4e^{\epsilon_{t}}}{n_{t}{\left(e^{\epsilon_{t}}-1\right)}^{2}}.$$

Conversely, if the transition is not updated in the current round, the model retains the previous Markov probability ${\tilde{M}}_{t}\left(i,j\right)$, which incurs an approximation error relative to the true transition probability $M_{t}^{*}\left(i,j\right)$, as the true probability $M_{t}^{*}\left(i,j\right)$ is not directly observable under the local differential privacy setting. We therefore approximate it using the aggregated perturbed estimate ${\hat{M}}_{t}\left(i,j\right)$, yielding

(14) $${\mathrm{Err}}_{\mathrm{app}}\left(i,j\right)={\left|{\hat{M}}_{t}\left(i,j\right)-{\tilde{M}}_{t}\left(i,j\right)\right|}^{2}.$$

To balance the two types of errors, we introduce a binary indicator variable $x_{ij}\in \left\{0,1\right\}$ for each transition $\left(f_{i}\to f_{j}\right)$, where $x_{ij}=1$ indicates that the transition is updated in the current round and $x_{ij}=0$ indicates that it remains unchanged. The significant transition selection problem can then be formulated as minimizing the total error over all transitions:

(15) $$\mathrm{Err}\left(t\right)\;=\;\sum_{i,j\in D_{\mathrm{tr}}}x_{ij}{\mathrm{Err}}_{\mathrm{upd}}\;+\;\sum_{i,j\in D_{\mathrm{tr}}}\left(1\;-\;x_{ij}\right){\mathrm{Err}}_{\mathrm{app}}\left(i,j\right).$$

Here, $D_{\mathrm{tr}}$ denotes the transition domain defined over the current set of leaf cells. After identifying significant transitions, the system updates and releases their corresponding probabilities, while non-significant transitions are retained unchanged.

### 4.4. Real-Time Trajectory Synthesis

After obtaining the two-layer adaptive grid $F_{t}$ and the corresponding mobility model $M_{t}$ at time *t*, the system performs real-time updates of synthetic trajectories. At each timestamp, the previous location $\left(x_{t-1},y_{t-1}\right)$ of a trajectory is remapped to the latest leaf cell $f_{j}$ according to the current grid $F_{t}$, ensuring that the trajectory state remains consistent with the dynamic spatial discretization.

For trajectories that are still active, the system samples the next state from the outgoing transition distribution $M_{t}\left(i,\cdot \right)$. If the sampled result corresponds to the exit state, the trajectory is terminated at time *t*. Otherwise, if the sampled result is a leaf cell $f_{j}$, the system uniformly samples a continuous coordinate $\left(x_{t},y_{t}\right)$ within the geometric region of $f_{j}$, and records it as the trajectory location at time *t*.

In addition to updating existing trajectories, the system maintains a target number of synthetic trajectories. At each timestamp, if the number of active trajectories falls below the target scale, the system samples new starting cells from the entry distribution $M_{t}\left(s^{\mathrm{in}},\cdot \right)$ and generates initial coordinates within the corresponding leaf cells to initiate new synthetic trajectories. Conversely, if the number of trajectories exceeds the target scale, a subset of trajectories is terminated according to the exit probabilities, so as to keep the synthetic trajectory population consistent with the scale of the real data.

### 4.5. Adaptive Privacy Budget Allocation

The *w*-event LDP constraint requires that the cumulative privacy budget within any sliding window of size *w* does not exceed ϵ. Therefore, before accessing the private transition event, AdaGrid-Syn determines the privacy budget for transition reporting using only mobility statistics that have been obtained by the end of timestamp t−1. Specifically, the system computes a trend statistic $T_{t}$ from the most recently available mobility model and its historical evolution, and then determines the privacy budget $\epsilon_{t}$ for the current transition report.

#### 4.5.1. Total Variation

To quantify the degree of change of the mobility model at timestamp *t* relative to its recent history, we construct a row-level total variation (TV) [47] metric for each origin cell *i*. At the beginning of timestamp *t*, the most recently available mobility model is ${\hat{M}}_{t-1}$. We further consider the average distribution of this row over the past κ timestamps, where κ denotes the window length: ${\bar{M}}_{t-1}\left(i,j\right)=\frac{1}{\kappa }\sum_{k=t-\kappa -1}^{t-2}{\hat{M}}_{k}\left(i,j\right)$. Here, *j* ranges over all legal transition targets of state *i* under the current transition domain. Based on this, the row-level total variation for cell *i* is defined as

(16) $${\mathrm{TV}}_{i}\left(t\right)=\frac{1}{2}\sum_{j}\left|{\hat{M}}_{t-1}\left(i,j\right)-{\bar{M}}_{t-1}\left(i,j\right)\right|.$$

If the dimensions of the legal transition domains $D_{t}$ and $D_{t-1}$ differ across timestamps due to adaptive grid refinement or merging, we reproject the mobility model from the previous timestamp onto the current grid using an area-based reprojection scheme, which is described in detail below.

To capture the global structural variation, we assign each origin cell a weight according to its relative transition activity. The weights are computed from the most recently available transition-frequency estimates ${\hat{C}}_{t-1}$:

(17) $$q_{t}\left(i\right)=\frac{\sum_{j:\left(i,j\right)\in D_{\mathrm{trans}}}{\hat{C}}_{t-1}\left(i,j\right)}{\sum_{k}\sum_{j:\left(k,j\right)\in D_{\mathrm{trans}}}{\hat{C}}_{t-1}\left(k,j\right)},f_{i}\in F_{t-1}.$$

$q_{t}\left(i\right)$ represents the relative frequency that a transition originates from leaf cell $f_{i}$.yielding the overall trend magnitude at timestamp *t*:

(18) $$T_{t}=\sum_{i}q_{t}\left(i\right)\;{\mathrm{TV}}_{i}\left(t\right).$$

The trend value $T_{t}\in \left[0,1\right]$ reflects the most recent structural variation available for the budget decision at timestamp *t*.

#### 4.5.2. Mobility Model Reprojection Under Adaptive Grids

In the two-layer adaptive grid structure, the leaf-cell partition may vary across timestamps, resulting in mobility model matrices with inconsistent dimensions. To compare transition structures and compute trend magnitudes in a unified state space, we perform mobility model reprojection whenever the grid changes, mapping the transition distribution defined on the old grid to the current grid.

Let $G_{\mathrm{old}}$ denote the old grid with $L_{\mathrm{old}}$ leaf cells, and $G_{\mathrm{new}}$ denote the new grid with $L_{\mathrm{new}}$ leaf cells. The mobility model on the old grid is represented as $M_{\mathrm{old}}\in R^{\left(L_{\mathrm{old}}+1\right)\times \left(L_{\mathrm{old}}+1\right)},$ where the last row and column correspond to the virtual enter/exit states.

Based on the geometric overlap between cells in the old and new grids, we first construct a reprojection matrix $W\in R^{L_{\mathrm{new}}\times L_{\mathrm{old}}}$, whose entries are defined as

(19) $$W_{ij}=\frac{\mathrm{Area}\left(c_{i}^{\mathrm{new}}\cap c_{j}^{\mathrm{old}}\right)}{\mathrm{Area}\left(c_{j}^{\mathrm{old}}\right)},$$

which indicates the proportion of probability mass of old cell *j* that should be mapped to new cell *i* according to their geometric overlap.

The matrix $M_{\mathrm{old}}$ is then decomposed according to the virtual-state structure into a leaf-to-leaf submatrix $A_{\mathrm{old}}$, a leaf-to-exit vector $b_{\mathrm{old}}$, and an enter-to-leaf vector $c_{\mathrm{old}}$. Linear reprojection is performed on the new grid as

(20) $$\begin{array}{cc} A_{\mathrm{new}} & =WA_{\mathrm{old}}W^{⊤},\\ b_{\mathrm{new}} & =Wb_{\mathrm{old}},\\ c_{\mathrm{new}} & =c_{\mathrm{old}}W^{⊤}, \end{array}$$

and the resulting components are reassembled to form the new mobility model matrix $M_{\mathrm{new}}$ that includes the virtual enter/exit states.

The reprojected matrix $M_{\mathrm{new}}$ may contain invalid transition probabilities caused by geometric stretching (e.g., long-distance jumps). These probabilities must be projected onto the legal set *S* in a probabilistic sense. Specifically, for each row *i*, let the unconstrained reprojected distribution be $\tilde{q}=M_{\mathrm{new}}\left(i,\cdot \right).$ Among all probability distributions supported only on transition domain $D_{\mathrm{tr}}$, we seek the one that is closest to $\tilde{q}$ in terms of KL divergence, i.e., the I-projection problem:

(21) $$\min_{p}\;\mathrm{KL}\left(p\;∥\;\tilde{q}\right)\;s.t.\left\{\begin{array}{c} p\left(j\right)=0,\;\forall j\notin D_{\mathrm{tr}},\\ \sum_{j}p\left(j\right)=1 \end{array}\right..$$

The closed-form solution is

(22) $$p\left(j\right)=\frac{\tilde{q}\left(j\right)}{\sum_{k:\;(i,k)\in S}\tilde{q}\left(k\right)},\;\left(i,j\right)\in S,$$

with p(j)=0 for all (i,j)∉S. That is, all probability mass on illegal transitions is removed, and the remaining mass on legal transitions is renormalized proportionally. This procedure discards physically infeasible transitions with minimum KL cost, while preserving the relative proportions among legal transitions and the row-stochastic property of the Markov model.

#### 4.5.3. Budget Allocation Under Mobility Variation

Before transition reporting at timestamp *t*, the system computes $T_{t}$ to determine the privacy budget for the current transition report. Let $\epsilon_{2}$ denote the total privacy budget allocated to all transition events.

To enhance sensitivity to abrupt structural changes under the *w*-event LDP constraint, we design a dynamically adjustable budget fraction $p_{t}\in \left[0,1\right]$ for each timestamp *t*, which determines the proportion of the available transition budget that can be consumed at the current time. Specifically, let the remaining available transition budget be

(23) $$\epsilon_{\mathrm{rm},t}=\epsilon_{2}-\sum_{\tau =t-w+1}^{t-1}\epsilon_{\tau }^{M}.$$

Since $T_{t}\in \left[0,1\right]$ reflects the rate of change in the overall transition distribution, more budget should be allocated when the system undergoes significant structural changes, while less budget is needed during stable periods. Accordingly, we define the following adaptive mapping:

(24) $$p_{t}=\min\left\{1-e^{-\alpha \cdot T_{t}},\;p_{\max}\right\},$$

where α>0 is a scaling factor controlling the overall budget consumption rate, and $p_{\max}$ specifies the maximum allowable budget fraction at a single timestamp. The privacy budget allocated to timestamp *t* is then given by $\epsilon_{t}=p_{t}\cdot \epsilon_{\mathrm{rm},t}.$

## 5. Theoretical Analysis

### 5.1. Privacy Analysis

**Theorem** **3.** *Under the proposed budget scheduling strategy, the mechanism presented in this paper satisfies w-event ϵ-LDP for each user.*

**Proof** **of Theorem 3.** In our framework, only the user-side local reporting operations directly access the raw trajectory data, while all subsequent procedures operate solely on perturbed data and thus constitute post-processing. According to Theorem 2, it suffices to show that the local reporting mechanisms satisfy ϵ-LDP within any sliding window of length *w*. Let $\epsilon_{1}$ denote the total privacy budget allocated to density events and $\epsilon_{2}$ denote the total budget allocated to transition events, where $\epsilon_{1}+\epsilon_{2}=\epsilon$.

For a single report at timestamp *t*, a user performs at most the following two operations:

1. Density reporting: This operation is performed once every *w* timestamps using the OUE mechanism, with privacy budget denoted by $\epsilon_{t}^{D}$. Therefore, density reporting either does not occur at timestamp *t* (i.e., $\epsilon_{t}^{D}=0$), or satisfies $\epsilon_{t}^{D}$-LDP.
2. Transition reporting: The transition is encoded as a unary vector and perturbed using the OUE mechanism with budget $\epsilon_{t}^{M}$, where $\epsilon_{t}^{M}=p_{t}\cdot \epsilon_{\mathrm{rm},t}$. Since OUE itself satisfies ϵ-LDP, this operation satisfies $\epsilon_{t}^{M}$-LDP.

The above two types of reports at timestamp *t* are sequentially composed. By Theorem 1, the overall reporting mechanism at timestamp *t* satisfies $\left(\epsilon_{t}^{M}+\epsilon_{t}^{D}\right)$-LDP. Let $\epsilon_{t}=\epsilon_{t}^{M}+\epsilon_{t}^{D}$.

We now consider the *w*-event constraint. Under the proposed budget scheduling strategy:

- Density reporting occurs at most once within each window, and the OUE perturbation for that report consumes a budget of $\epsilon_{1}$, i.e., $\sum_{i=t-w+1}^{t}\epsilon_{i}^{D}=\epsilon_{1}$;
- For any sliding window of length *w*, denoted by [t−w+1,t], the total budget used for transition reporting satisfies $\sum_{i=t-w+1}^{t}\epsilon_{i}^{M}=\sum_{i=t-w+1}^{t}p_{i}\cdot \epsilon_{\mathrm{rm},i}\leq \epsilon_{2}$.
  Therefore, for any window of length *w*, we have
  (25) $$\sum_{i=t-w+1}^{t}\epsilon_{i}=\sum_{i=t-w+1}^{t}\left(\epsilon_{i}^{D}+\epsilon_{i}^{M}\right)\leq \epsilon_{1}+\epsilon_{2}=\epsilon .$$
  Hence, the proposed mechanism satisfies *w*-event ϵ-LDP for each user. □

### 5.2. Utility Analysis

We consider a system operating over consecutive timestamps t=0,1,…,T−1, with window size *w*.

At timestamp *t*, let the current set of leaf cells be $F_{t}$, whose cardinality is $|F_{t}|$. Since the system allows only self-loops, neighbor transitions, and enter/exit events, the size of the transition domain grows linearly with the number of leaf cells, i.e., $|S_{t}\left|=O(9\right|F_{t}\left|\right).$

1. User-side computational cost:
  (a) Transition reporting:
    Each true transition is encoded as a unary vector of dimension $|S_{t}|$, and each bit is perturbed once using OUE. The resulting complexity is $O(|S_{t}\left|\right)=O(|F_{t}\left|\right)$.
  (b) Density reporting (once per window, amortized):
    Every *w* timestamps, OUE is applied to a vector of dimension $|F_{t}|$. The amortized cost per timestamp is $O(|F_{t}|/w)$.
2. Server-side computational cost:
  Let $n_{t}$ be the number of users reporting at timestamp *t*, and let $n_{t}^{\mathrm{syn}}$ denote the number of synthetic trajectories that are still active.
  The server first computes the number of significant transitions, deviation metrics, and the privacy budget fraction $p_{t}$ allocated to the transition matrix. This step requires traversing the current transition domain $S_{t}$, and thus has complexity $O(|S_{t}\left|\right)=O(9|F_{t}\left|\right).$
  Aggregating the OUE transition reports from users incurs a complexity of $O(n_{t}\cdot |S_{t}|)=O(n_{t}\cdot |F_{t}\left|\right).$ Performing significant updates based on the aggregated OUE results depends only on the transition domain and has complexity $O(|S_{t}\left|\right)=O(9|F_{t}\left|\right)$.
  At the end of each window, the server aggregates leaf-cell densities using OUE and reselects refinement levels for all coarse cells, with total complexity $O(|F_{t}|),$ which amortizes to $O(|F_{t}|/w)$ per timestamp.
  During real-time synthesis, at timestamp *t* the server extends all unfinished synthetic trajectories and adjusts the total number of trajectories to match the target scale. For each active synthetic trajectory, the next state is sampled according to the current Markov matrix and neighborhood structure, resulting in a complexity of $O(n_{t}^{\mathrm{syn}})$. Adjusting the number of trajectories incurs an additional cost of $O(n-n_{t}^{\mathrm{syn}})$.
3. Communication overhead:
  At timestamp *t*, communication primarily occurs between real users and the server, dominated by the upload of perturbed transition reports. Each active user uploads one OUE-perturbed transition encoding vector, whose length is proportional to the transition domain size. Thus, the communication cost per user is $O(9|F_{t}|)$, and the total uplink cost is $O(9\cdot n_{t}\cdot |F_{t}|)$.

In addition, users upload a density vector once at the end of each window. The amortized communication cost per timestamp for density reporting is $O(n_{t}\cdot |F_{t}|/w)$. At window boundaries, the server broadcasts the updated grid structure or coarse cell refinement levels. Such messages depend only on the number of coarse cells, which remains fixed throughout execution. Therefore, the amortized per-timestamp communication overhead for these broadcasts is constant and negligible compared with the uplink cost.

## 6. Experiments

In this section, we compare the proposed method with state-of-the-art approaches to validate the effectiveness of AdaGrid-Syn.

### 6.1. Experiment Settings

#### 6.1.1. Datasets

We conduct experiments on one real-world trajectory dataset and two synthetic trajectory datasets. The statistical characteristics of these datasets are reported in Table 1.

The T-Drive dataset [48] contains real GPS trajectories collected from 10,357 taxis in Beijing over one week. Following the setting in [21], we select a high-density region within the Fifth Ring Road, and discretize the time axis into 886 time slots with a granularity of 10 min.

The SanJoaquin and Oldenburg datasets are generated using the Brinkhoff moving object generator [49]. Specifically, SanJoaquin is synthesized based on the road network of the city of SanJoaquin, containing 1200 timestamps, with an initial number of 10,000 users and 300 new users added at each timestamp. Oldenburg is generated using the same mechanism but based on the road network of the city of Oldenburg; it contains 450 timestamps, with an initial number of 20,000 users and 1000 new users added at each timestamp. For both synthetic datasets, the interval between adjacent timestamps is approximately 15 s.

#### 6.1.2. Baselines

To the best of our knowledge, RetraSyn [19] is the state-of-the-art real-time trajectory synthesis scheme that satisfies strict *w*-event LDP. It includes two variants corresponding to different privacy resource allocation strategies, namely RetraSyn^b^ and RetraSyn^p^. In addition, we incorporate two representative methods from the LDP-IDS [29] framework, LBA and LPA. However, LDP-IDS is tailored for histogram publishing scenarios, making it inherently unsuitable for direct use in trajectory publication. Therefore, for fair comparison, we adapt both methods following the modification strategy adopted in [19].

1. RetraSyn^b^ adopts a budget-splitting strategy that distributes the overall privacy budget across timestamps, selectively updates significantly changed transitions using noisy statistics, and synthesizes trajectories based on a global Markov model that incorporates enter/exit events.
2. RetraSyn^p^ adopts a user-splitting strategy that no longer divides the budget along time; instead, it samples a subset of active users at each timestamp, allowing them to perturb their reports using the full ϵ, thereby achieving lower noise sensitivity when the active user population fluctuates.
3. LBA adopts a uniform privacy budget allocation scheme, in which the budget associated with non-sampled points is absorbed by the sampled points.
4. LPA adopts a distribution approach similar to LBA, but instead of allocating the privacy budget, it distributes across the user population.

### 6.2. Evaluation Metrics

To systematically evaluate the effectiveness of synthetic trajectories from the perspectives of both dynamic modeling and global structural preservation, we design eight metrics from two aspects: streaming consistency and historical structure fidelity. Streaming metrics characterize the statistical quality of synthetic trajectories during the generation process, while historical metrics focus on the fidelity of accumulated structural characteristics.

#### 6.2.1. Streaming Metrics

- Density Error. This metric evaluates whether the synthetic trajectories can reproduce the time-varying spatial visit distribution. Let $P_{t}\left(T\right)$ and $P_{t}\left(T_{\mathrm{syn}}\right)$ denote the grid density distributions of the real trajectory set T and the synthetic trajectory set $T_{\mathrm{syn}}$ at timestamp *t*, respectively. Density error is defined as
  (26) $$DE=\frac{1}{T}\sum_{t=1}^{T}\mathrm{JSD}\left(P_{t}\left(T\right),P_{t}\left(T_{\mathrm{syn}}\right)\right).$$
  Here, JSD(·) denotes the Jensen–Shannon divergence between two distributions.
- Spatial–Temporal Query Error. This metric measures the ability of synthetic trajectories to support count-based queries. Each query corresponds to a rectangular region and a time window, and we count the visits of real and synthetic trajectories within the specified range. Let $Q_{\mathrm{real}}^{\left(k\right)}$ and $Q_{\mathrm{syn}}^{\left(k\right)}$ denote the real and synthetic counts for the *k*-th query, respectively. To avoid error amplification when the real count is small, we introduce a stabilizer sb, set to 1% of the all-time average visit count in the real system. The overall spatial–temporal query error is defined as
  (27) $$QE=\frac{1}{N}\sum_{k=1}^{N}\frac{\left|Q{\left(T\right)}^{\left(k\right)}-Q{\left(T_{\mathrm{syn}}\right)}^{\left(k\right)}\right|}{\max\;\left(Q{\left(T_{\mathrm{syn}}\right)}^{\left(k\right)},\;\mathrm{sb}\right)}.$$
  In experiments, we set N=100.
- Transition Error. This metric measures whether the migration patterns between regions are preserved. Let $M_{t}$ and ${\hat{M}}_{t}$ denote the real and synthetic transition matrices at timestamp *t*, respectively. Transition error is defined as
  (28) $$TraE=\frac{1}{T}\sum_{t=1}^{T}\mathrm{JSD}\left(M_{t},{\hat{M}}_{t}\right).$$
- Hotspot NDCG. This metric evaluates the ability of synthetic trajectories to preserve spatial hotspot regions. We use the normalized discounted cumulative gain (NDCG) to measure whether the ranking of the top-*h* most visited regions within a randomly selected time window is accurately reproduced. In experiments, we set h=10 and report the average over 100 randomly sampled windows.
- Pattern F1. This metric evaluates whether mobility patterns are preserved by the synthetic trajectories. Let $S_{\mathrm{real}}$ and $S_{\mathrm{syn}}$ be the top-*N* pattern sets mined from the real and synthetic trajectories within a time window, respectively. Precision and recall are defined as
  (29) $$P=\frac{|S_{\mathrm{real}}\cap S_{\mathrm{syn}}|}{|S_{\mathrm{syn}}|},\;R=\frac{|S_{\mathrm{real}}\cap S_{\mathrm{syn}}|}{|S_{\mathrm{real}}|}.$$
  The Pattern F1 score is then given by
  (30) $$\mathrm{PatternF}1=\frac{2PR}{P+R}.$$
  In experiments, we set N=100.

#### 6.2.2. Historical Metrics

- Kendall’s Tau. This metric measures the consistency of regional popularity rankings. Let ${\mathrm{rank}}_{\mathrm{real}}\left(C_{i}\right)$ and ${\mathrm{rank}}_{\mathrm{syn}}\left(C_{i}\right)$ denote the ranks of cell $C_{i}$ in the real and synthetic trajectories, respectively. Let $N_{c}$ and $N_{d}$ be the numbers of concordant and discordant pairs. Kendall’s tau is defined as
  (31) $$\tau =\frac{N_{c}-N_{d}}{\frac{\left|C|(|C|-1\right)}{2}}.$$
- Length Error. This metric examines whether the trip-scale structure is consistent. Let $L_{\mathrm{real}}$ and $L_{\mathrm{syn}}$ denote the trajectory length distributions of real and synthetic trajectories, respectively. The error is defined as
  (32) $$LE=\mathrm{JSD}(L_{\mathrm{real}},L_{\mathrm{syn}}).$$
- Trip Error. This metric evaluates whether the origin–destination structure is preserved. Let T(T) and $T(T_{\mathrm{syn}})$ denote the joint origin–destination probability distributions of real and synthetic trajectories, respectively. The error is defined as
  (33) $$TriE=\mathrm{JSD}\left(T\left(T\right),T\left(T_{\mathrm{syn}}\right)\right).$$

### 6.3. Overall Performance

We first compare the overall utility performance of AdaGrid-Syn with the baselines on three datasets, The total privacy budget is fixed at ϵ=1. For each dataset, we run all the methods five times using different random seeds and report the averaged results. As summarized in Table 2, AdaGrid-Syn consistently achieves overall utility improvements across multiple datasets for both Streaming metrics and historical metrics. This consistent performance gain mainly stems from two aspects.

On the one hand, the proposed two-layer adaptive grid dynamically adjusts spatial resolution according to the actual access density and structural complexity. It enables finer-grained modeling in high-activity regions while avoiding excessive partitioning in sparse areas, which would otherwise amplify noise, thereby yielding more reliable statistical estimates under the same privacy constraints.

On the other hand, the dynamic privacy budget scheduling mechanism adaptively allocates the limited privacy budget based on the degree of mobility pattern variation. This strategy allows the system to devote more privacy budget to timestamps with significant structural changes, while reducing unnecessary privacy consumption during relatively stable periods, thus maintaining high overall synthesis quality over long-term continuous operation.

In addition, the identical Length Error of 0.6931 for LBA and LPA corresponds to the maximum Jensen–Shannon divergence, ln2. This is caused by the lack of entering/quitting modeling in the adapted baselines, which leads to a strong mismatch between the real and synthetic cumulative trajectory distance distributions.

### 6.4. Ablation Study and Parameter Variation

#### 6.4.1. Impact of Allocation Strategies

We first evaluate the impact of privacy budget allocation strategies between density events ($\epsilon_{1}$) and transition events ($\epsilon_{2}$) on utility. Due to space limitations, we report three representative metrics on T-Drive dataset. The total privacy budget is fixed at ϵ=1, while the allocation ratio of $\epsilon_{1}$ to $\epsilon_{2}$ is varied from 1:9 to 9:1 such that $\epsilon_{1}+\epsilon_{2}=\epsilon$. As illustrated in Figure 2, the utility performance of AdaGrid-Syn achieves its global peak at a ratio of ϵ_1_:ϵ_2_ = 3:2. All three evaluation metrics exhibit a consistent synergistic trend, which can be categorized into three distinct phases:

- In the regime where $\epsilon_{1}$ is low, the privacy budget allocated to density events is insufficient to suppress the injected noise. Consequently, AdaGrid-Syn fails to perform accurate adaptive grid refinement, leading to significant structural distortion in the synthetic trajectories.
- As $\epsilon_{1}$ increases, the capability of AdaGrid-Syn to capture the underlying true data patterns improves steadily. The experimental results confirm that the performance across all three metrics reaches its optimum when the budget is balanced at a ratio of ϵ_1_:ϵ_2_ = 3:2.
- When the ratio exceeds 3:2, further increasing $\epsilon_{1}$ results in an insufficient budget for transition events ($\epsilon_{2}$). In this phase, the critical mobility characteristics of the original trajectories are obscured by excessive noise perturbations, causing a collective degradation in utility across all metrics.

Based on the aforementioned empirical analysis, we set the default allocation ratio for density and transition events to 3:2 to ensure an optimal balance between spatial refinement and mobility feature preservation.

For transition events, in addition to the proposed adaptive privacy budget allocation strategy, we also implement two baseline methods, namely Uniform and Sample. In the Sample strategy, the entire privacy budget for each window is allocated to the first timestamp within the window. Specifically, all users report their data once every *w* timestamps, each time using a privacy budget of $\frac{2\epsilon }{5}$. In contrast, the Uniform strategy evenly distributes the total privacy budget across all timestamps in the window, such that each timestamp is assigned a budget of $\frac{2\epsilon }{5w}$.

We compare these two baseline strategies with our adaptive approach, and the results are shown in Figure 3. Overall, the adaptive allocation strategy demonstrates stronger robustness and achieves better overall utility with relatively stable advantages. Only on the SanJoaquin dataset does the Sample strategy obtain the lowest transition error. This phenomenon can be attributed to the relatively stable evolution of trajectory streams in the SanJoaquin dataset, under which periodic model updates remain a competitive solution. Moreover, under the adaptive strategy, the allocated privacy budget is not necessarily fully consumed, which may lead to partial underutilization of privacy resources. This observation is consistent with recent work [19,31], which indicates that data-independent allocation strategies can be sufficient for certain analysis tasks. However, although the Sample strategy achieves favorable transition error on the SanJoaquin dataset, its performance on other evaluation metrics degrades noticeably. In contrast, the adaptive allocation strategy maintains superior utility across different datasets and multiple evaluation metrics.

#### 6.4.2. Impact of Adaptive Grid

To isolate the contribution of the proposed adaptive grid, we construct a fixed grid variant, denoted as FixedGrid-Syn. Specifically, FixedGrid-Syn uniformly partitions the entire spatial domain into a fixed k×k grid, which remains unchanged throughout the trajectory stream. To avoid confounding the effect of adaptive gird with the number of spatial states, *k* is selected according to the average number of leaf cells generated by AdaGrid-Syn.

Let $\bar{L}=\frac{1}{T}\sum_{t=1}^{T}\left|F_{t}\right|$ denote the average number of leaf cells over all timestamps. We choose $k=\arg\min_{m\in N}\left|m^{2}-\bar{L}\right|,$ so that the number of cells in the fixed grid is as close as possible to the average spatial-domain size of AdaGrid-Syn. FixedGrid-Syn uses the same budget allocation schedule as AdaGrid-Syn. Table 3 reports the results on four utility metrics under the same total privacy budget of ϵ=1.

As shown in Table 3, AdaGrid-Syn consistently achieves better utility than FixedGrid-Syn across the three datasets. The results indicate that adaptively allocating spatial resolution according to local mobility characteristics can better preserve spatial distributions and transition patterns than using a fixed spatial partition under the same downstream processing procedure.

#### 6.4.3. Impact of Privacy Budget ϵ

This section compares the synthesis utility of AdaGrid-Syn with baselines under different privacy budget settings. Due to space constraints and the fact that LBA is not fully tailored for trajectory publication, we limit our comparison to AdaGrid-Syn and the two variants of RetraSyn. As shown in Figure 4, with the increase in the privacy budget, all methods exhibit a general trend of performance improvement across most utility metrics, which is consistent with the fundamental property of local differential privacy that the magnitude of injected noise decreases as the privacy budget increases. Moreover, AdaGrid-Syn consistently outperforms its competitors across all evaluated metrics.

We further observe that, on the Kendall’s Tau metric, the two variants of RetraSyn exhibit pronounced non-monotonic fluctuations under different privacy budgets. This phenomenon mainly stems from the high sensitivity of Kendall’s Tau to the relative ranking of location visit frequencies. In the two baseline methods, which rely on relatively static spatial modeling and uniform noise injection, subtle frequency differences among hotspot locations are more susceptible to perturbation by local differential privacy noise. In contrast, AdaGrid-Syn is able to preserve the relative structure of hotspot regions more effectively, yielding a much smoother variation in ranking consistency with respect to the privacy budget.

#### 6.4.4. Impact of Window Size *w*

To analyze the impact of the window size *w* on the utility of trajectory synthesis, we conduct experiments on the T-Drive and SanJoaquin datasets by varying *w* from 10 to 50. The experimental results are shown in Figure 5.

Overall, as the window size *w* increases, all compared methods exhibit varying degrees of performance degradation across the evaluated metrics. This is because a larger window contains more timestamps that need to be protected, which in turn reduces the amount of resources allocated to each individual timestamp. On the T-Drive dataset, the performance degradation with respect to *w* is relatively mild. In contrast, AdaGrid-Syn consistently achieves the lowest error under all window configurations, and its error growth rate is significantly smaller than that of the two RetraSyn variants, demonstrating stronger robustness to changes in the window size. On the SanJoaquin dataset, the impact of increasing *w* is more pronounced, especially for query error and trip error. This can be attributed to the longer trajectories and broader spatial coverage in SanJoaquin, where a larger window is more likely to introduce structural mixing effects, thereby amplifying the influence of noise on statistical estimation. Nevertheless, AdaGrid-Syn still consistently outperforms RetraSyn^b^ and RetraSyn^p^ under all window settings.

In summary, although increasing the window size *w* inevitably leads to certain utility degradation, AdaGrid-Syn is able to maintain stable and consistent performance advantages across different values of *w*, indicating its strong adaptability to variations in the window parameter.

### 6.5. Computational Efficiency

To analyze the computational efficiency of the proposed framework, we decompose the overall pipeline into the following four major sub-procedures: (i) User-side Computation, (ii) Two-Layer Adaptive Grid Structure, (iii) Mobility Model Construction, and (iv) Real-Time Trajectory Synthesis. Other operations, such as privacy budget allocation, incur negligible computational overhead compared with the above sub-procedures and are therefore omitted from the efficiency evaluation.

It is worth noting that the Two-Layer Adaptive Grid Structure is not triggered at every timestamp, but is executed only once at the end of each time window of length *w*. To fairly characterize its contribution to the overall runtime, we amortize the computational cost incurred within a window over the corresponding *w* timestamps when reporting the average processing time per timestamp.

As shown in Table 4, the real-time trajectory synthesis component dominates the overall computational cost. This is because trajectory synthesis requires generating new locations and performing data size adjustment with a time complexity of $O(T_{\mathrm{syn}})$. Overall, the average processing time per timestamp of our approach is significantly lower than the duration between consecutive timestamps, demonstrating its practicality for real-time trajectory publishing scenarios.

## 7. Conclusions

This paper proposes a real-time trajectory publishing framework under local differential privacy constraints for trajectory streams generated in MCS environments, termed AdaGrid-Syn. By introducing a two-layer adaptive spatial grid structure, AdaGrid-Syn dynamically adjusts spatial resolution according to trajectory access density, thereby improving modeling accuracy in complex and non-uniform scenarios while preserving privacy guarantees.

In dynamic spatial domains, the proposed framework jointly models trajectory density and mobility patterns, and further incorporates an adaptive privacy budget allocation strategy, enabling a flexible balance between privacy consumption and data utility at different evolution stages. Experimental results demonstrate that AdaGrid-Syn consistently outperforms existing baseline methods across multiple utility metrics under various privacy budgets, window sizes, and datasets, and exhibits strong robustness to dataset characteristics and parameter variations. Overall, AdaGrid-Syn provides an effective and practical solution for real-time trajectory publishing in mobile crowdsensing under local differential privacy.

## Figures and Tables

**Figure 1:**
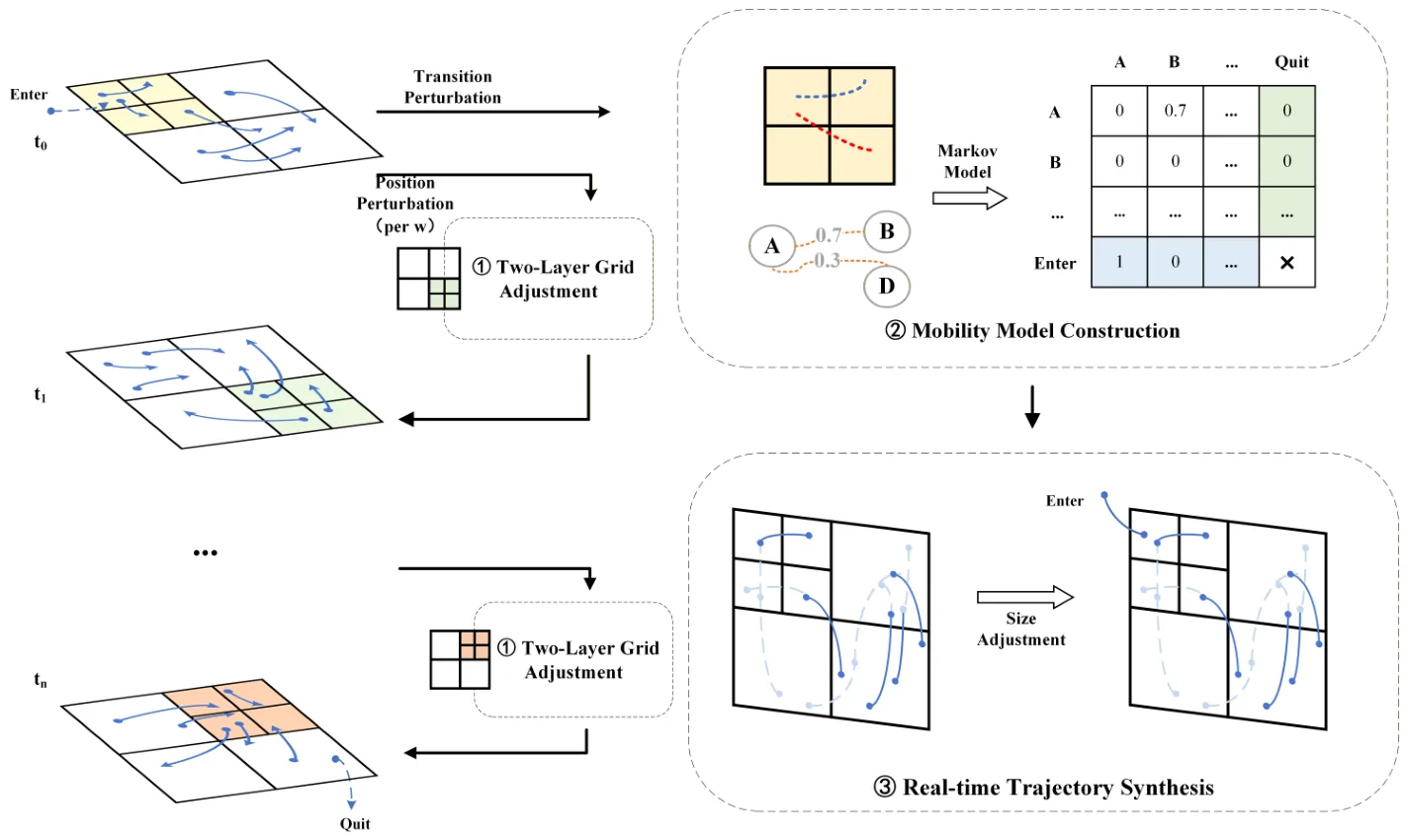
Overview of the proposed AdaGrid-Syn framework.

**Figure 2:**
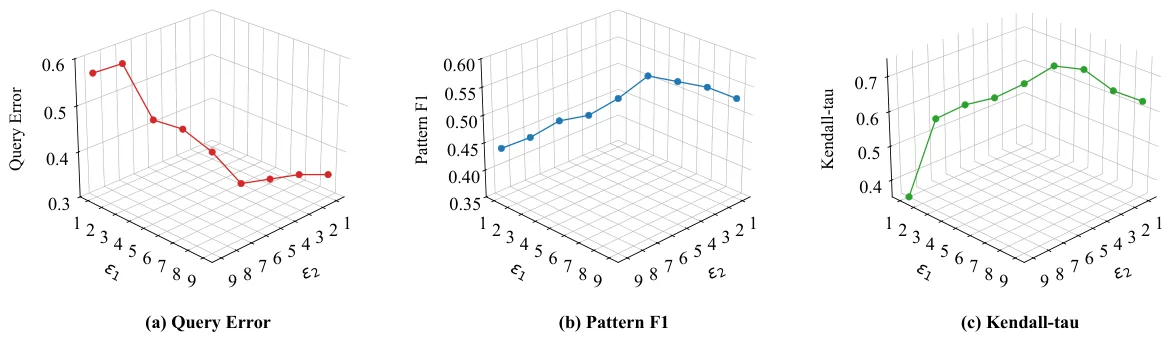
Impact of allocation strategies between $\epsilon_{1}$ and $\epsilon_{2}$. Smaller values are better for query error and larger values are better for the other metrics.

**Figure 3:**
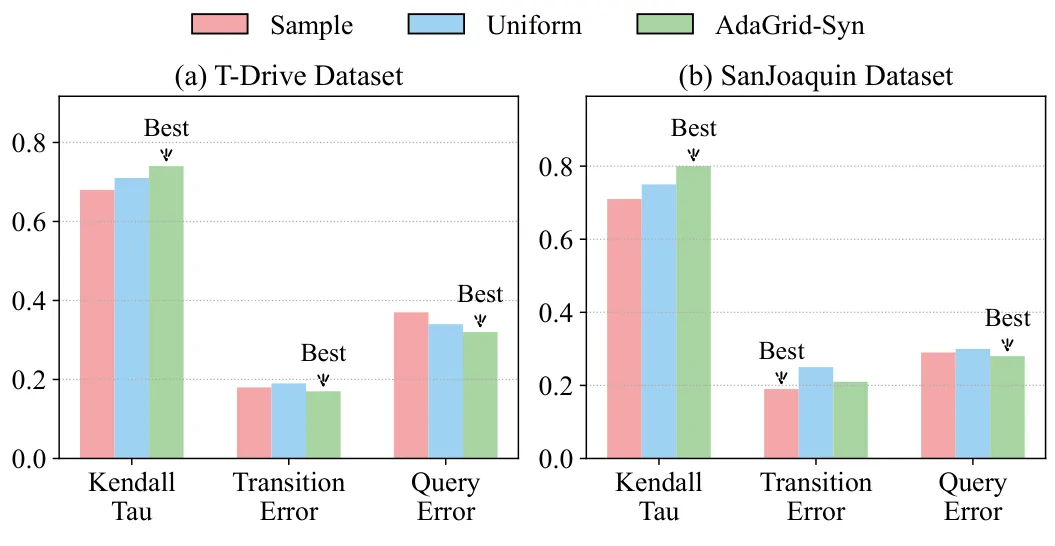
Impact of allocation strategy of transition events. Larger values are better for Kendall-tau and smaller values are better for the other metrics.

**Figure 4:**
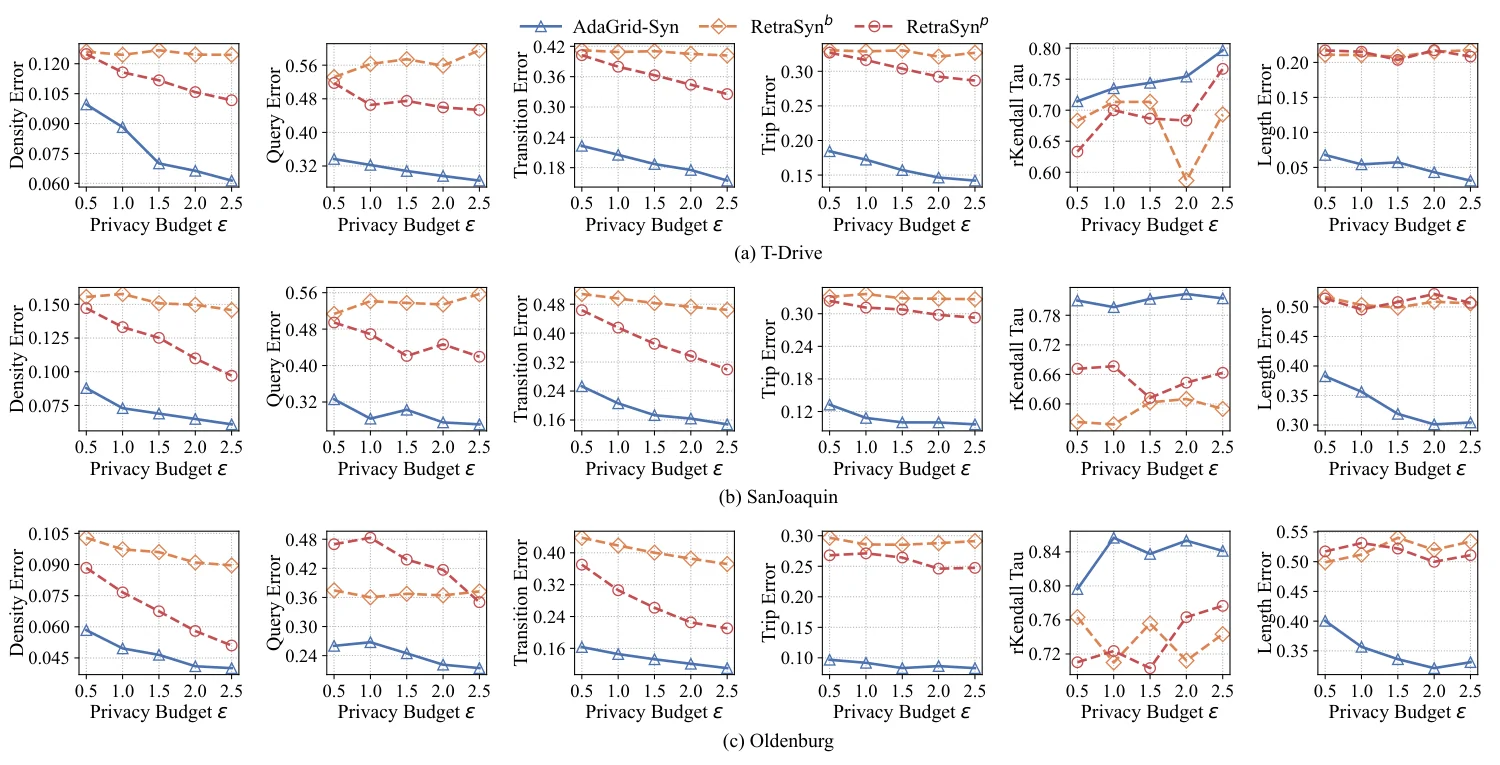
Impact of ϵ. Larger values are better for Kendall-tau and smaller values are better for the other metrics.

**Figure 5:**
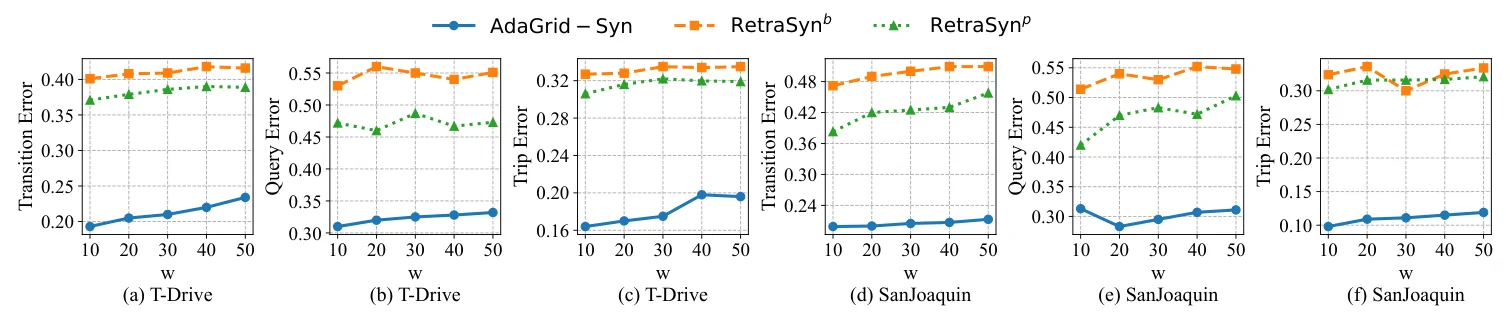
Impact of window size *w*.

**Table 1:** Statistics of datasets.

Dataset	Size	Avg Length	Timestamps	Sampling Interval
T-Drive	232,640	13.61	886	10 min
SanJoaquin	370,000	53.83	1200	15 s
Oldenburg	470,000	69.20	450	15 s

**Table 2:** Utility comparison. For Kendall-tau, Hotspot NDCG and Pattern F1, larger values are better; for the remaining metrics, lower values are better.

Dataset	Method	Metrics							
		Density	Query	Transition	Hotspot	Pattern	Kendall	Length	Trip
		Error	Error	Error	NDCG	F1	-Tau	Error	Error
T-Drive	LBA	0.2314	1.1520	0.5467	0.2321	0.1354	0.2251	0.6931	0.4215
	LPA	0.6132	1.5658	0.6379	0.2217	0.1957	0.0567	0.6931	0.6864
	RetraSyn^b^	0.1275	0.5646	0.4083	0.6353	0.4775	0.7175	0.2137	0.3275
	RetraSyn^p^	0.1149	0.4736	0.3794	0.6975	0.4964	0.7025	0.2149	0.3153
	AdaGrid-Syn	**0.0858**	**0.3236**	**0.2052**	**0.7876**	**0.5764**	**0.7371**	**0.0565**	**0.1718**
SanJoaquin	LBA	0.6743	0.9863	0.6872	0.1884	0.0526	0.4054	0.6931	0.6877
	LPA	0.6043	1.4521	0.6654	0.1365	0.1851	0.1845	0.6931	0.6823
	RetraSyn^b^	0.1535	0.5476	0.4965	0.6098	0.5465	0.5575	0.5067	0.3410
	RetraSyn^p^	0.1329	0.4687	0.4164	0.8121	0.5636	0.6787	0.4963	0.3138
	AdaGrid-Syn	**0.0718**	**0.2842**	**0.2073**	**0.8954**	**0.7464**	**0.7974**	**0.3573**	**0.1067**
Oldenburg	LBA	0.0983	0.7213	0.4553	0.6463	0.2154	0.2437	0.6931	0.2689
	LPA	0.3972	1.0695	0.5267	0.3964	0.3483	0.1554	0.6931	0.6145
	RetraSyn^b^	0.0956	0.3658	0.4175	0.6873	0.4785	0.7123	0.5101	0.2812
	RetraSyn^p^	0.0776	0.4832	0.3074	0.7937	0.5162	0.7253	0.5279	0.2693
	AdaGrid-Syn	**0.0487**	**0.2683**	**0.1483**	**0.8345**	**0.7601**	**0.8563**	**0.3573**	**0.0928**

Bold values indicate the best results.

**Table 3:** Impact of adaptive grid.

Dataset	Method	Density Error	Query Error	Transition Error	Trip Error
T-Drive	FixedGrid-Syn	0.1345	0.4532	0.3432	0.2534
	AdaGrid-Syn	**0.0858**	**0.3236**	**0.2052**	**0.1718**
SanJoaquin	FixedGrid-Syn	0.1134	0.3734	0.3127	0.1843
	AdaGrid-Syn	**0.0718**	**0.2842**	**0.2073**	**0.1067**
Oldenburg	FixedGrid-Syn	0.0625	0.3529	0.2875	0.1653
	AdaGrid-Syn	**0.0487**	**0.2683**	**0.1483**	**0.0928**

Bold values indicate the best results.

**Table 4:** Component efficiency of AdaGrid-Syn. We report the average processing time per timestamp in seconds.

Component	T-Drive	SanJoaquin	Oldenburg
User-side Computation	0.0015	0.0039	0.0172
Two-Layer Adaptive Grid Structure	0.0016	0.0042	0.0188
Mobility Model Construction	0.0009	0.0011	0.0018
Real-Time Trajectory Synthesis	0.1032	0.3269	1.6281
Total	0.1056	0.3321	1.6480

## Data Availability

The experimental data used to support the findings of this study are available from the corresponding author upon request.
